# Supplementary material for: Regulating protein corona on nanovesicles by glycosylated polyhydroxy polymer modification for efficient drug delivery
Source: Nat Commun. 2024 Feb 7;15:1159. doi: 10.1038/s41467-024-45254-7 (PMC10850157; doi:10.1038/s41467-024-45254-7)
Supplement: Supplementary file 1 — Supplementary Information [file 41467_2024_45254_MOESM1_ESM.docx]

Supplementary Information

Regulating Protein Corona on Nanovesicles by Glycosylated Polyhydroxy Polymer Modification for Efficient Drug Delivery

Yunqiu Miao^1,2^, Lijun Li^1,3^, Ying Wang^1,3^, Jiangyue Wang^1^, Yihan Zhou^1^, Linmiao Guo^1^, Yanqi Zhao^1,3^, Di Nie^1^, Yang Zhang^2^, Xinxin Zhang^*1,3,4^, Yong Gan^*1,3,5^

^1^State Key Laboratory of Drug Research, Shanghai Institute of Materia Medica, Chinese Academy of Sciences, Shanghai 201203, China

^2^Shanghai Tenth People’s Hospital, School of Medicine, Tongji University, Shanghai 200072, China

^3^University of Chinese Academy of Sciences, Beijing 100049, China

^4^Shandong Laboratory of Yantai Drug Discovery, Bohai Rim Advanced Research Institute for Drug Discovery, Yantai, Shandong 264117, China

^5^NMPA Key Laboratory for Quality Research and Evaluation of Pharmaceutical Excipients, National Institutes for Food and Drug Control, Beijing 100050, China

E-mail: Xinxin Zhang (xinxinzhang@simm.ac.cn); Yong Gan (ygan@simm.ac.cn)


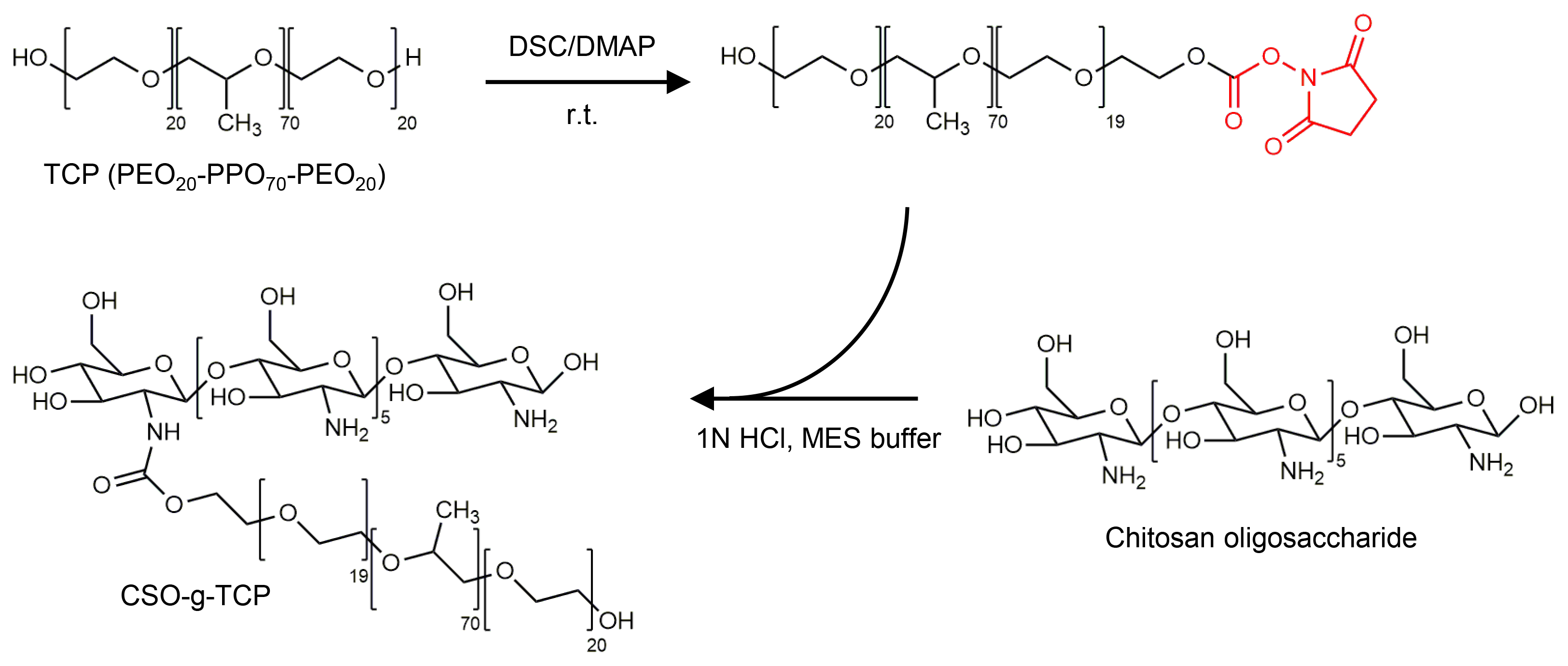


**Supplementary Fig. 1** The synthetic procedures of CSO-g-TCP.


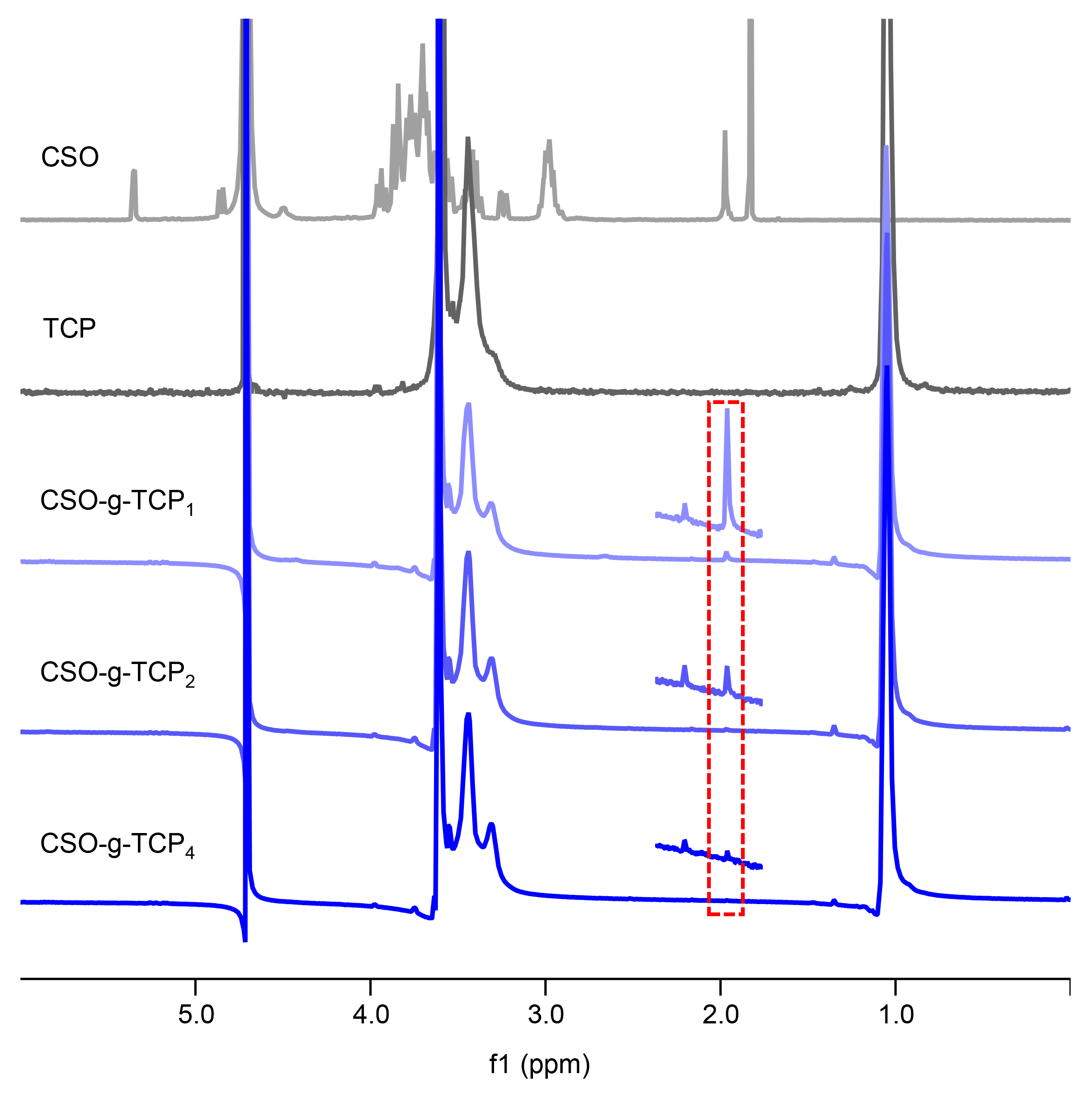


**Supplementary Fig. 2** ^1^H-NMR spectra of CSO, TCP, CSO-g-TCP_1_, CSO-g-TCP_2_ and CSO-g-TCP_4_ dissolved in deuterated water detected by nuclear magnetic resonance spectrometer (AVANCE III 400, Bruker Biospin, Switzerland). The spectrums of CSO-g-TCP_1_, CSO-g-TCP_2_ and CSO-g-TCP_4_ present similar signals for the ethylene protons of PEO segments and methyl protons of PPO segments at 3.6 and 1.0 ppm, respectively, as compared with TCP. For CSO chain of CSO-g-TCP, the hydrogen protons of glycosyl group at 3.0-4.0 ppm were observed both in CSO and CSO-g-TCP. The peak around 2.0 ppm showed different densities in CSO-g-TCP indicated the different grafting ratios of CSO and TCP. The grafting ratio of CSO and TCP was calculated through the molar mass of the remaining acetyl (δ_2.2_) in CSO and the molar mass of methyl protons (δ_1.0_) in PPO segments.


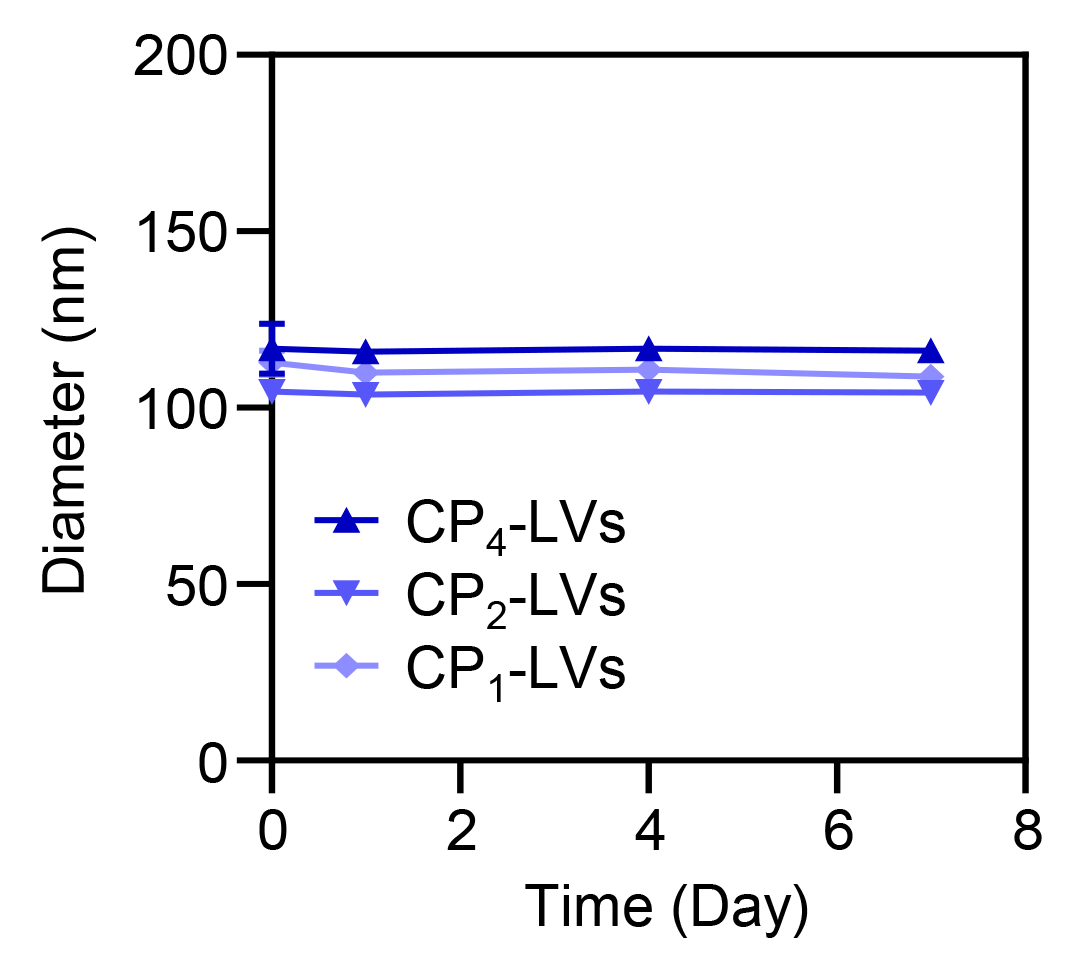


**Supplementary Fig. 3** The size of CP-LVs in simulated physiological environment over 7 days. Data are displayed as the mean ± SD (n = 3 independent samples).


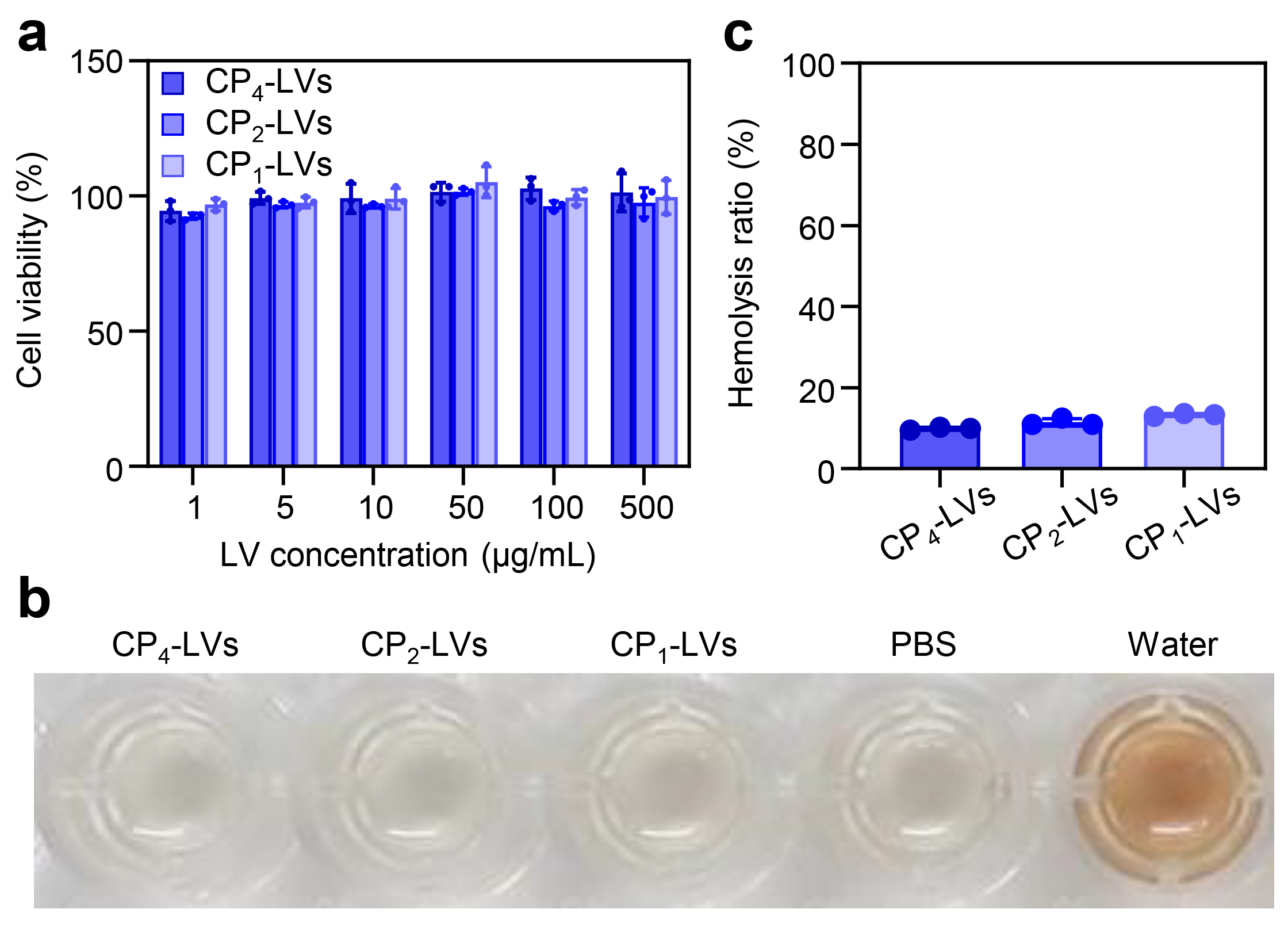


**Supplementary Fig. 4** **a** The cell viability of HUVECs treated with CP-LVs at different concentrations. **b** In vitro hemolysis assay of CP-LVs. **c** The hemolysis ratio of CP-LVs. Data are displayed as the mean ± SD (n = 3 independent samples).


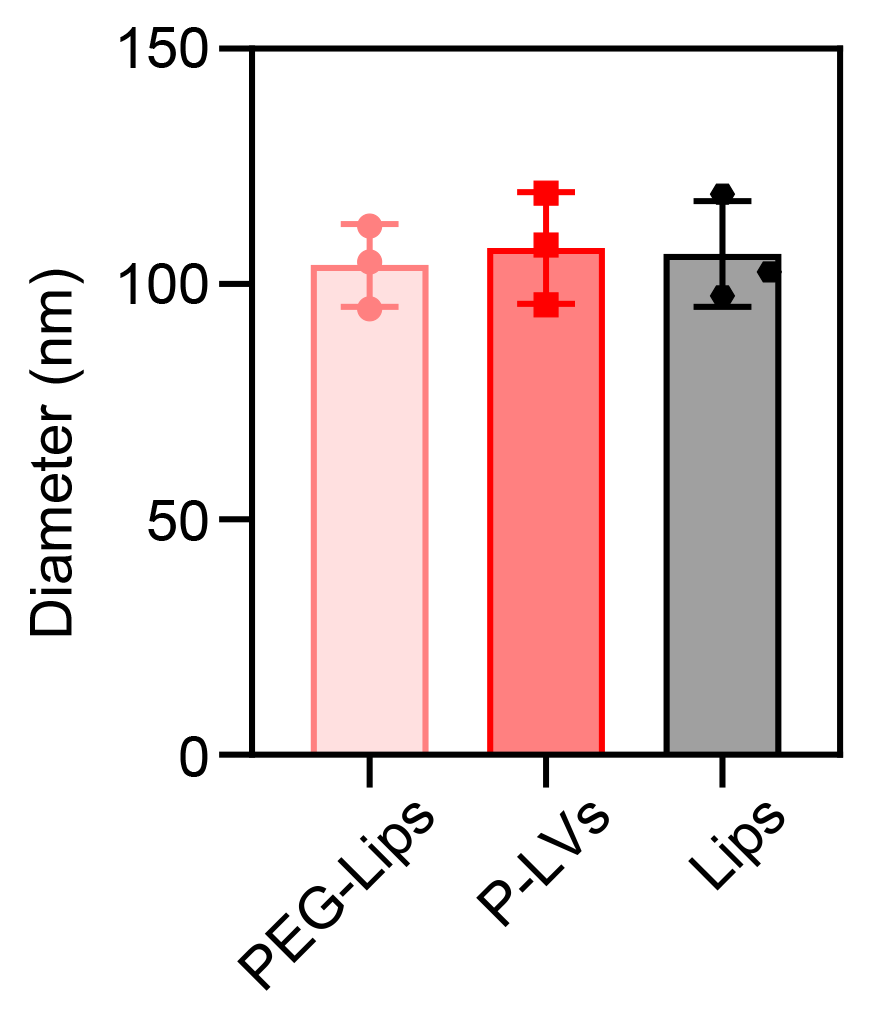


**Supplementary Fig. 5** The average size of PEG-Lips, P-LVs and Lips. Data are displayed as the mean ± SD (n = 3 independent samples).


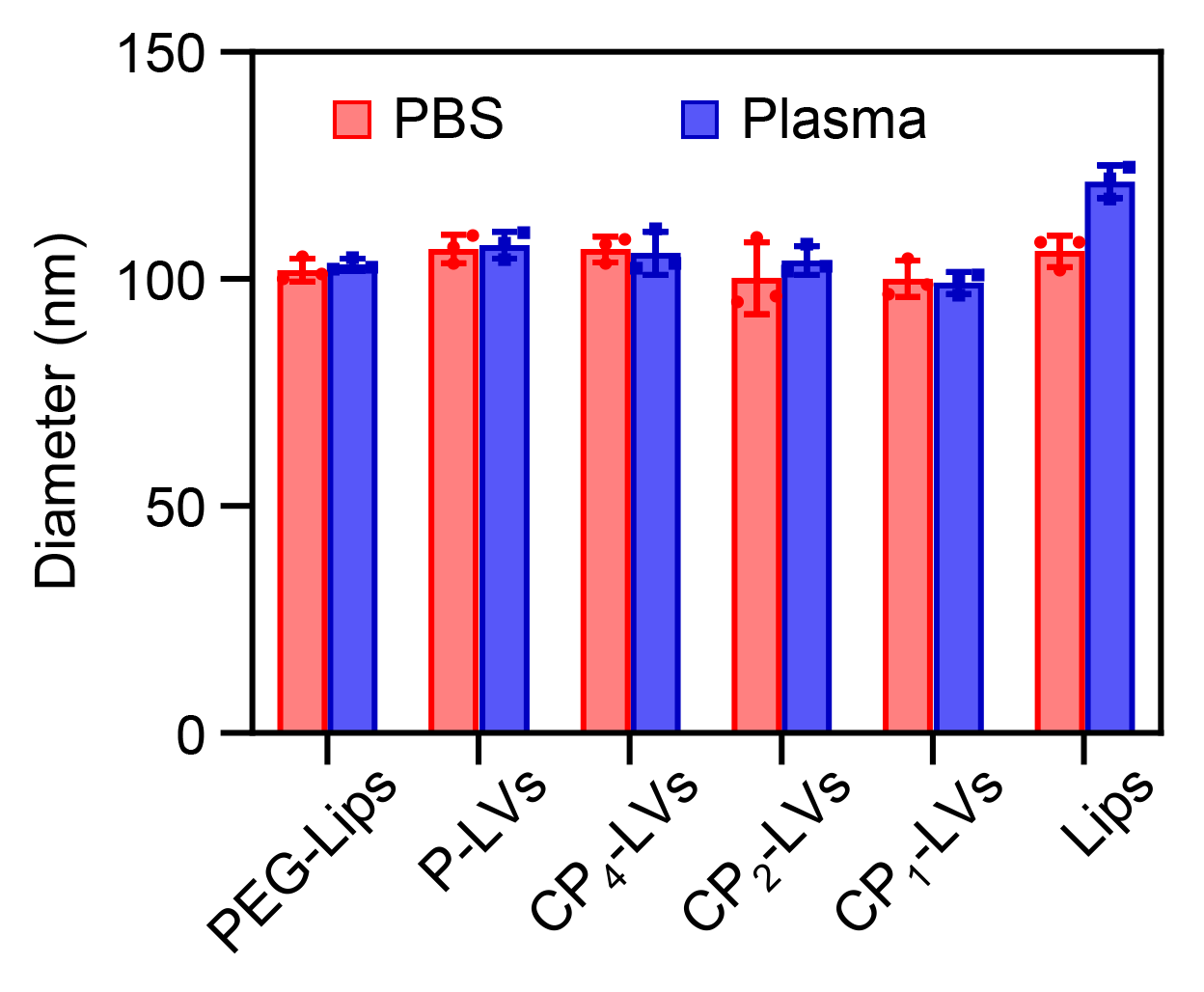


**Supplementary Fig. 6** The changes in size of PEG-Lips, P-LVs, CP-LVs and Lips with or without plasma incubation. Data are displayed as the mean ± SD (n = 3 independent samples).


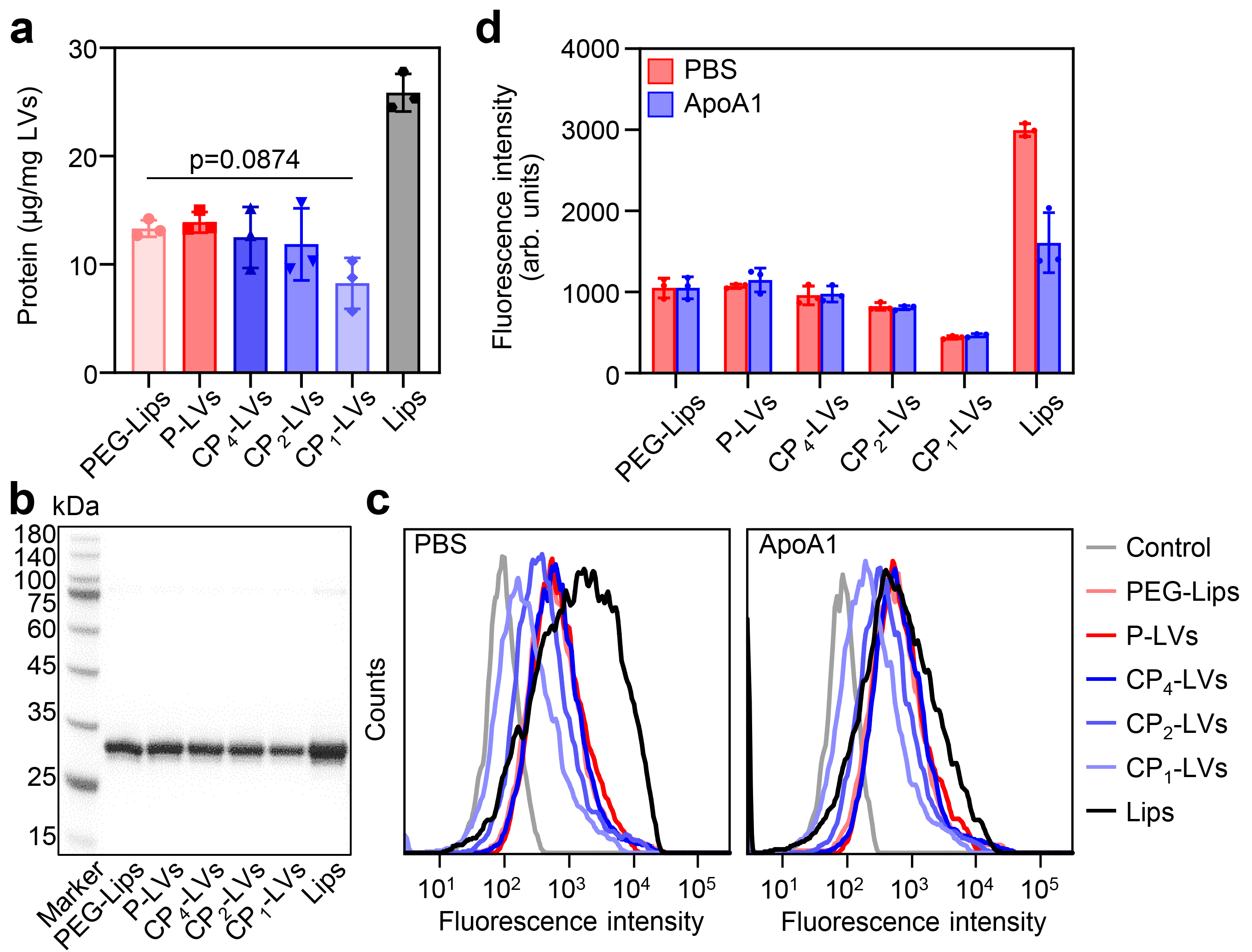


**Supplementary Fig. 7 a** Quantification of ApoA1 adsorbed to the nanovesicle surface determined by BCA assay. The data are displayed as the mean ± SD (One-way ANOVA, n = 3 independent samples). **b** Qualitative molecular composition of the adsorbed protein layer on the nanovesicles by SDS-PAGE. (n = 3 independent samples). **c** Representative flow cytometric histograms of nanovesicles with or without ApoA1 adsorption within J774 cells. (n = 3 independent samples). **d** Fluorescence intensity of nanovesicles with different protein coronas within J774 cells. The data are displayed as the mean ± SD (n = 3 independent samples).


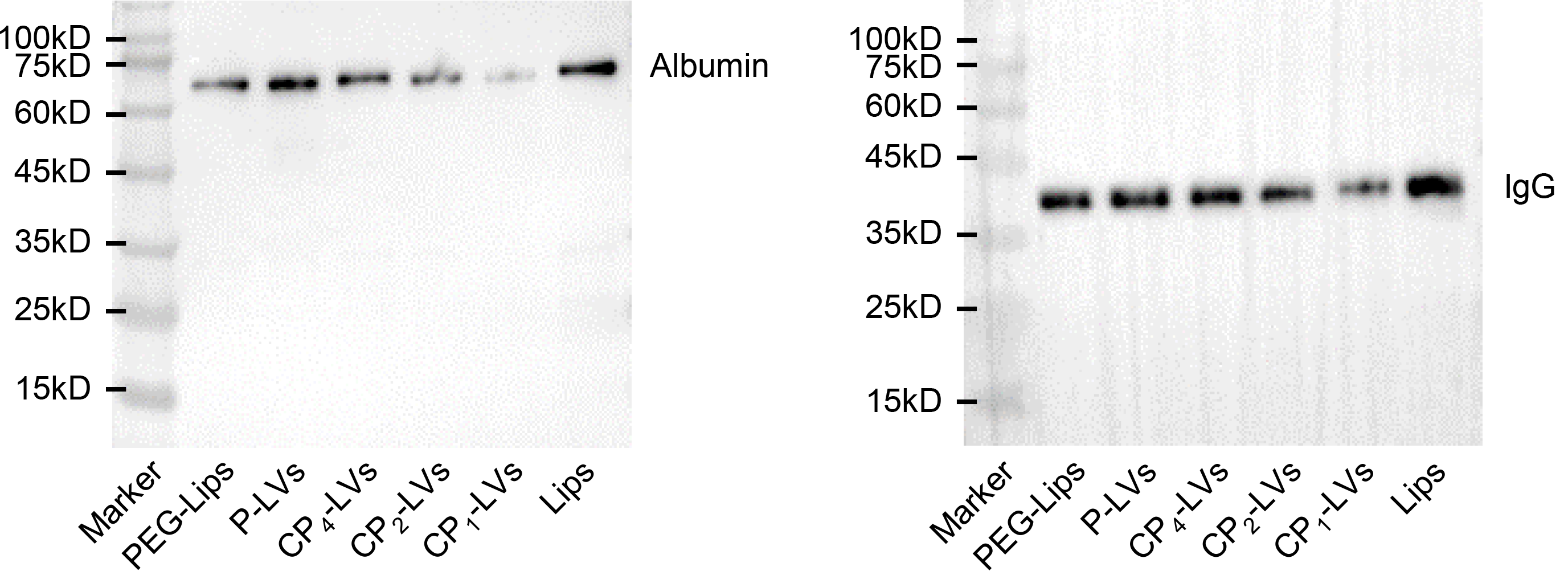


**Supplementary Fig. 8** Western blotting of albumin and IgG adsorbed on nanovesicles. (n = 3 independent samples).


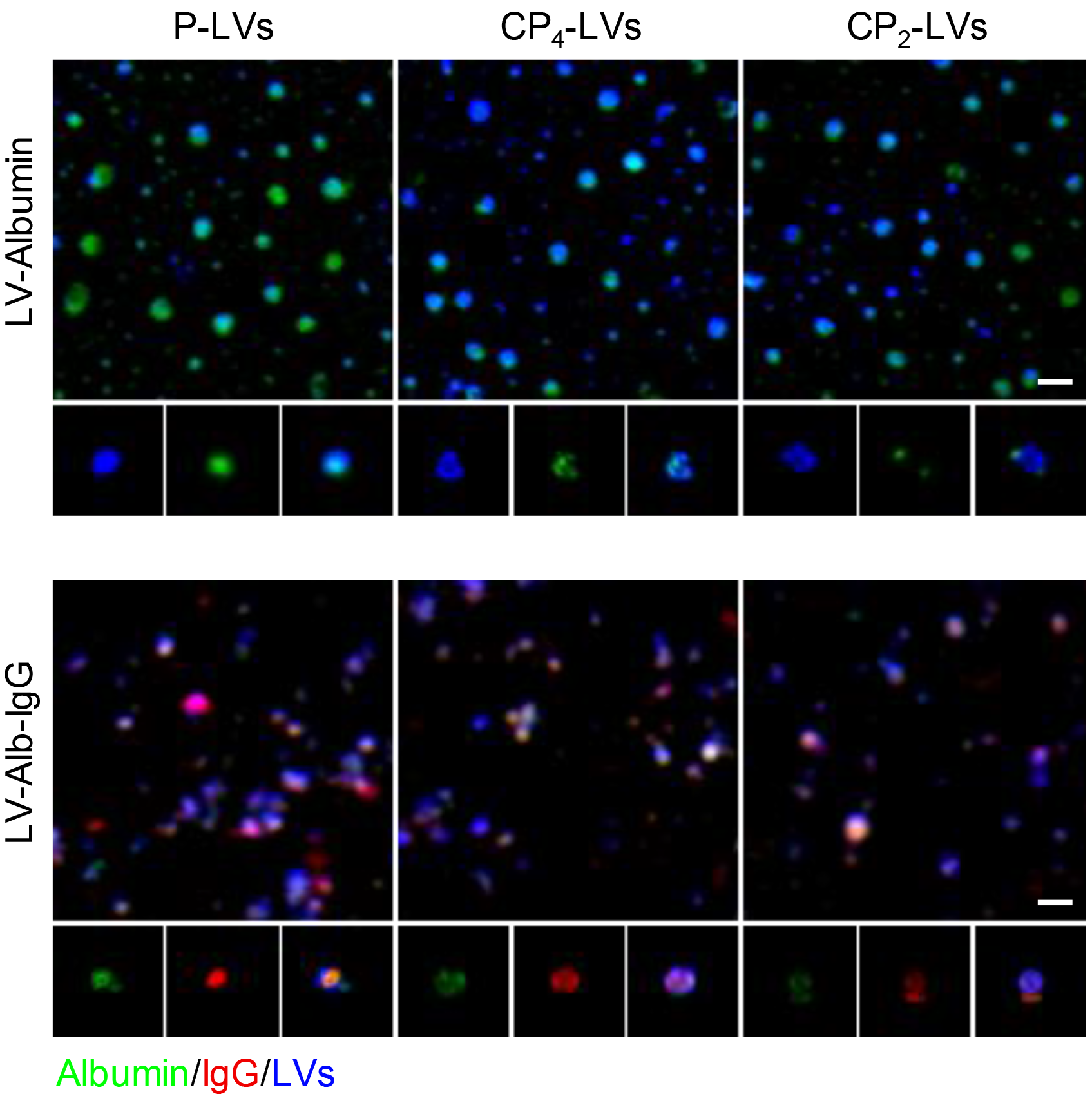


**Supplementary Fig. 9** Representative confocal images of P-LVs, CP_4_-LVs and CP_2_-LVs incubated with albumin (upper panel) and IgG solution (lower panel). Blue: nanovesicles; Green: Albumin; Red: IgG. The magnified images of single liposomes were shown under the confocal images. Scale bar: 100 nm. (n = 3 independent samples).


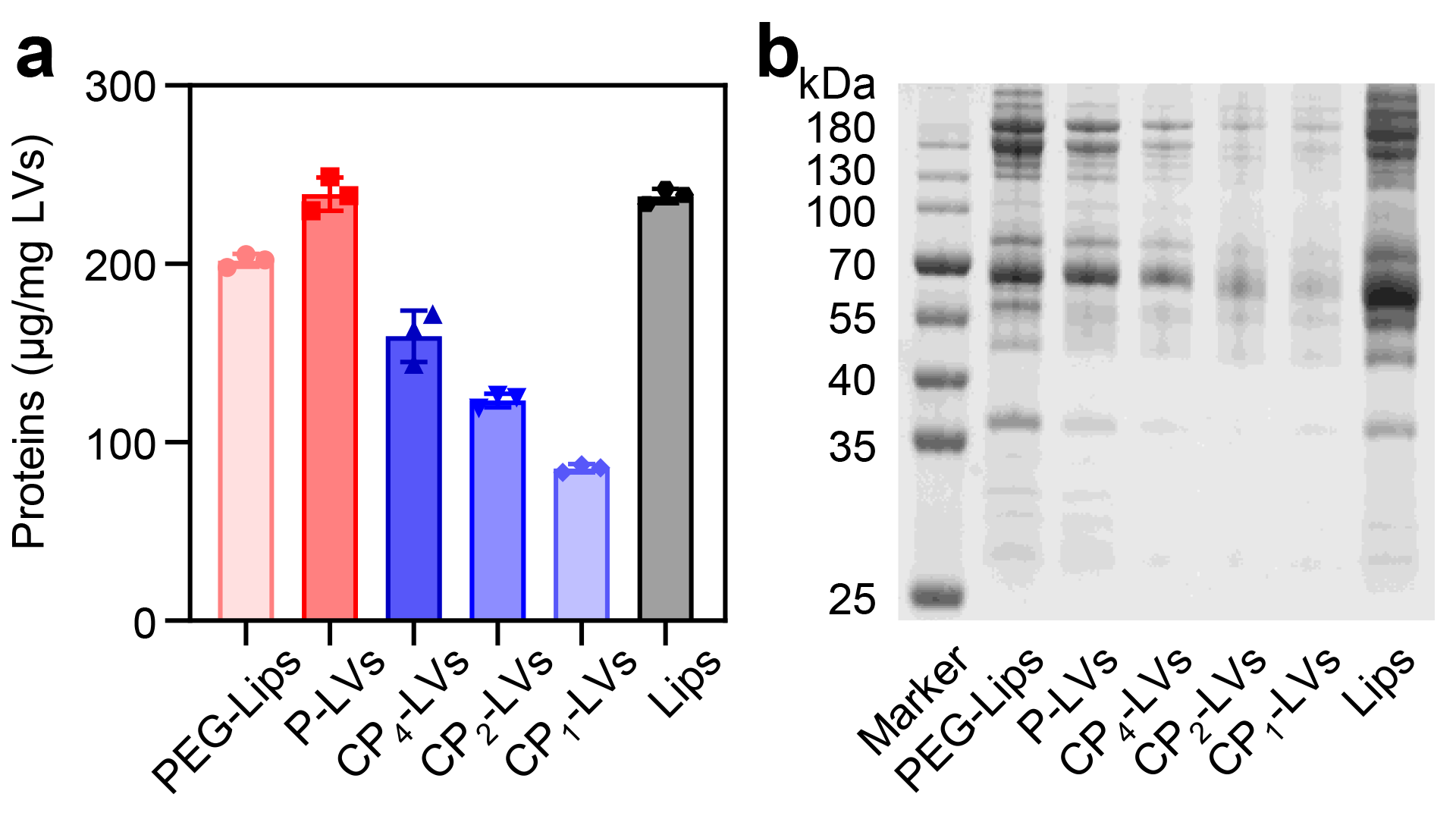


**Supplementary Fig. 10 a** Quantification of proteins adsorbed to the nanovesicles recovered from plasma in mice preinjected with nanovesicles. The data are displayed as the mean ± SD (n = 3 independent samples). **b** Qualitative molecular composition of the adsorbed protein layer on nanovesicles recovered from plasma in mice preinjected with nanovesicles. Data are displayed as the mean ± SD (n = 3 independent samples).


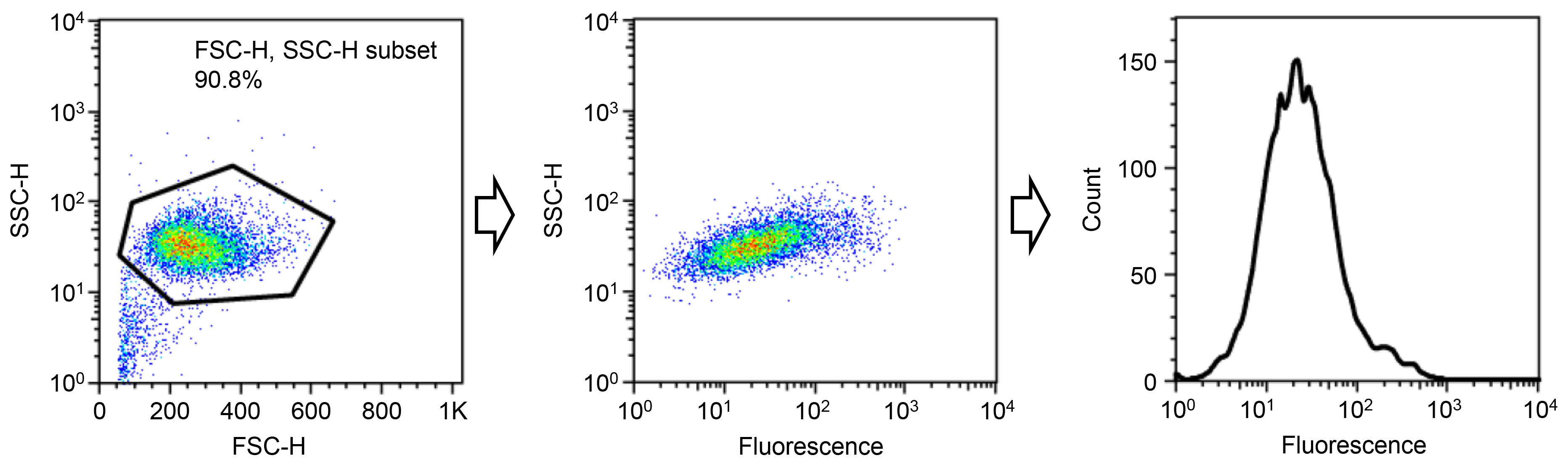


**Supplementary Fig. 11** Gating strategy (presented using J774 cells) to determine the nanovesicle internalization in cells.


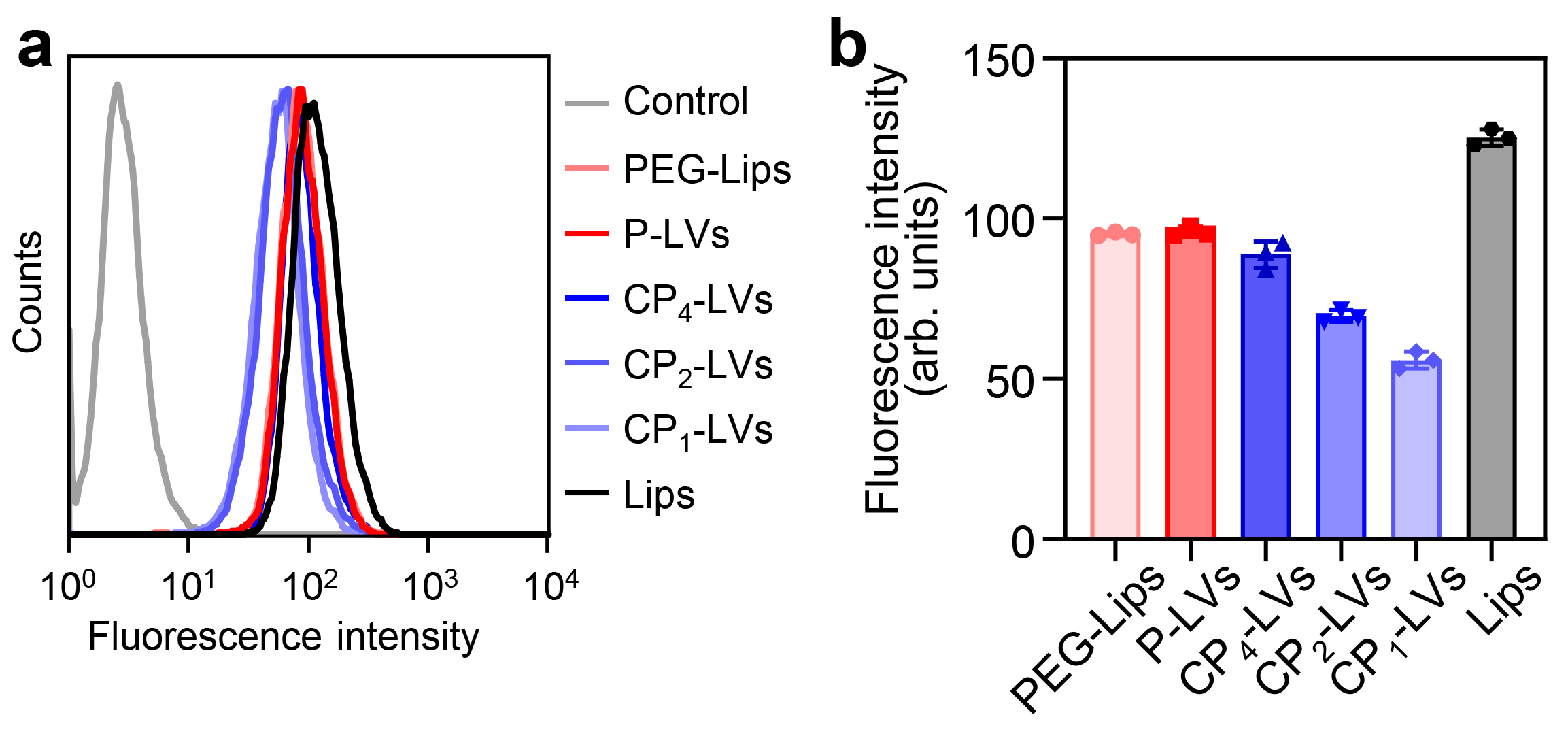


**Supplementary Fig. 12 a** Representative flow cytometric histograms and **b** mean fluorescence intensity of DiO-labeled nanovesicles within J774 cells. Data are displayed as the mean ± SD (n = 3 independent samples).


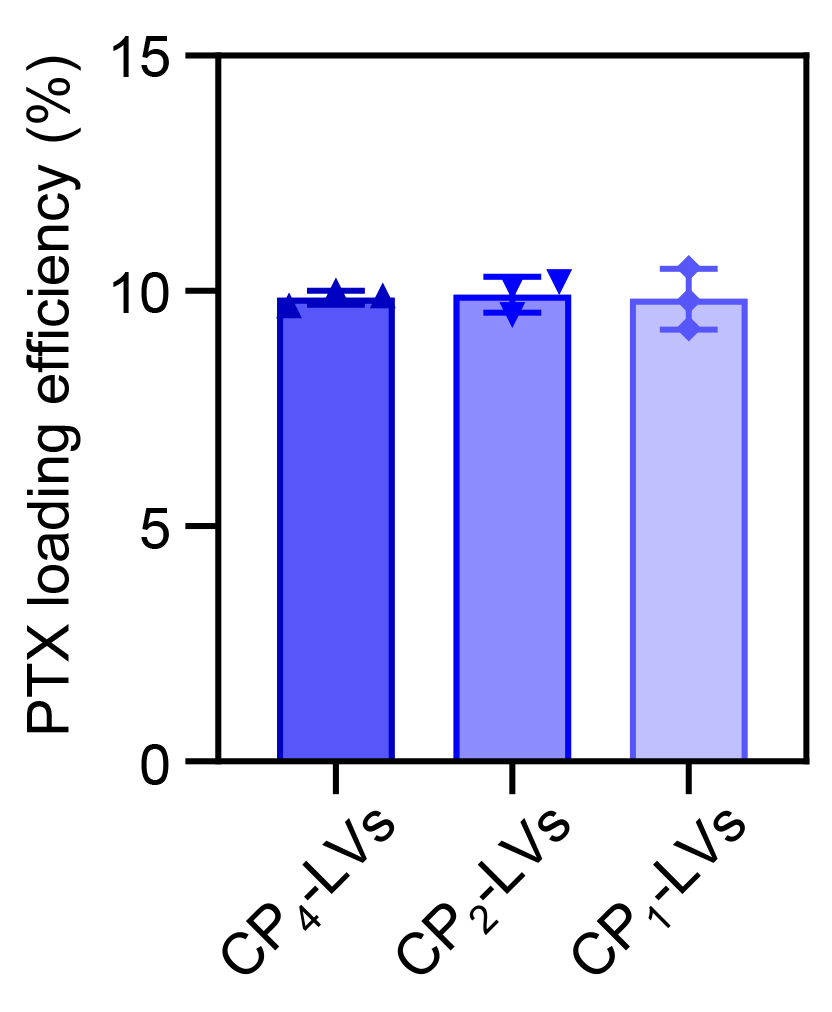


**Supplementary Fig. 13** Paclitaxel (PTX) loading efficiency of CP_4_-LVs, CP_2_-LVs and CP_1_-LVs. Data are displayed as the mean ± SD (n = 3 independent samples).


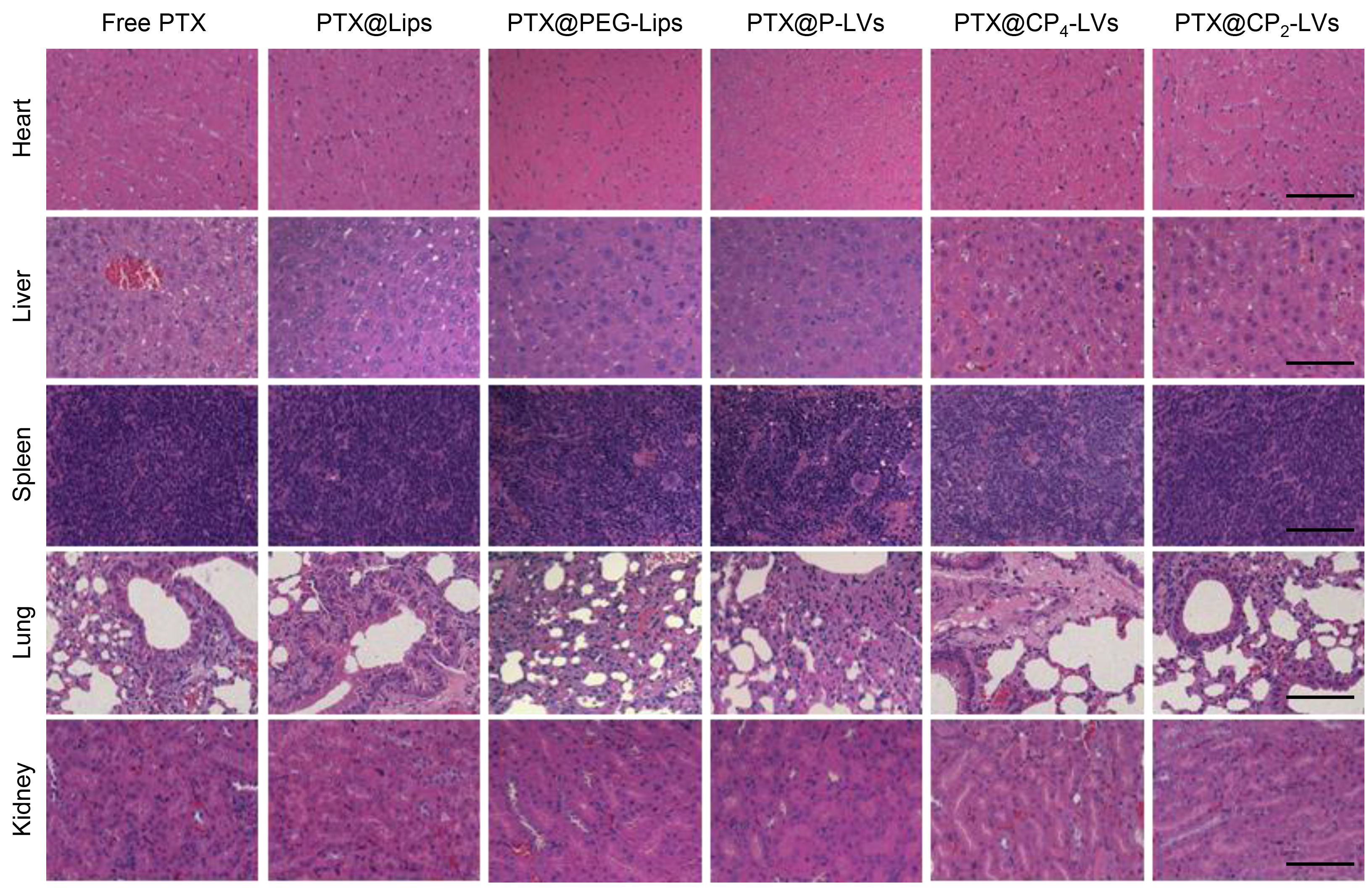


**Supplementary Fig. 14** Representative H&E-stained histological sections of main organs after treatments. Scale bar = 100 μm.


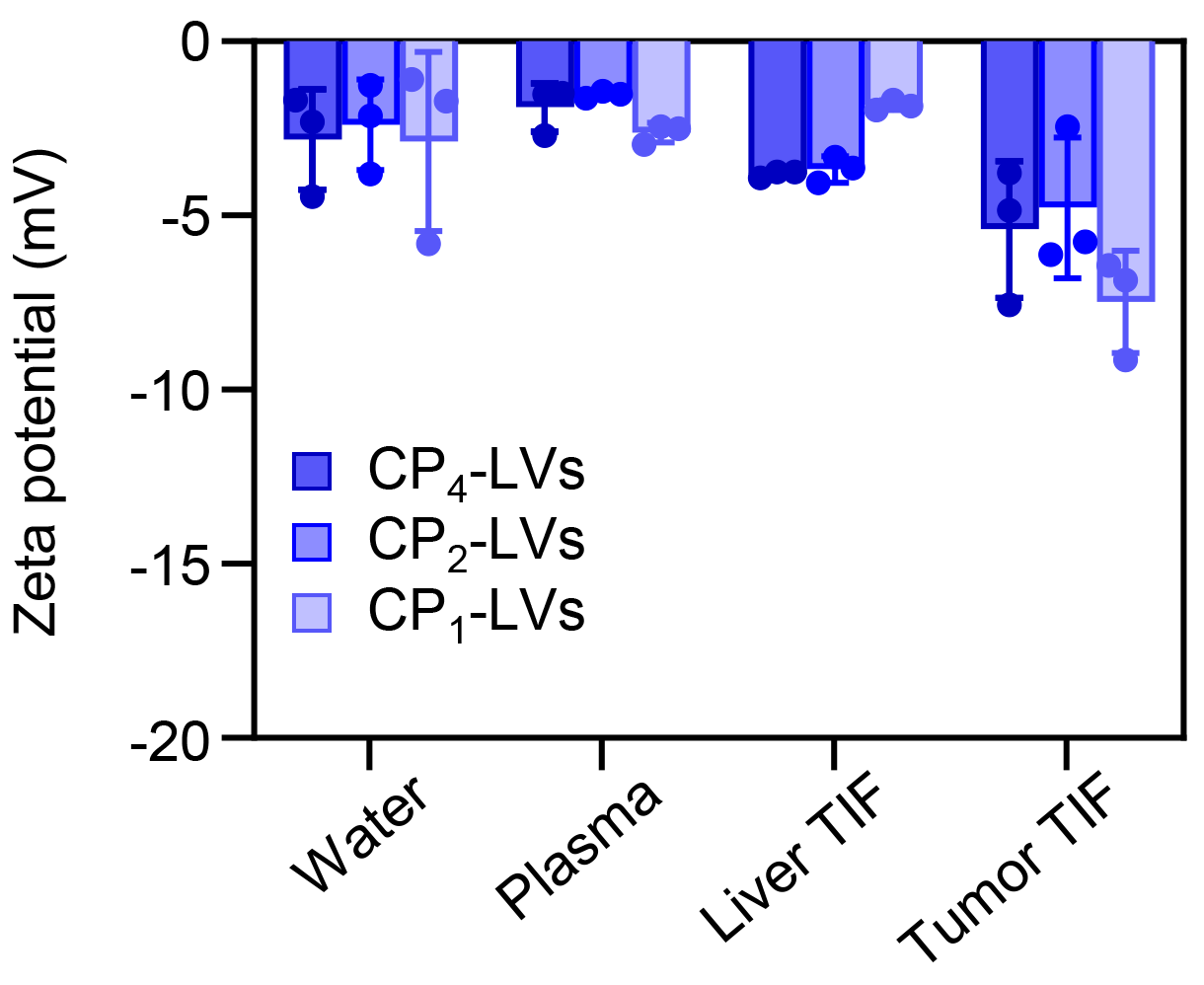


**Supplementary Fig. 15** The zeta potential of nanovesicles incubating with plasma, liver TIF and HeLa tumor TIF, compared with that of nanovesicles dispersed in water. The data are presented as mean ± SD (n=3 independent samples).
