# Supplementary material for: Regulating protein corona on nanovesicles by glycosylated polyhydroxy polymer modification for efficient drug delivery
Source: Nat Commun. 2024 Feb 7;15:1159. doi: 10.1038/s41467-024-45254-7 (PMC10850157; doi:10.1038/s41467-024-45254-7)
Supplement: Supplementary file 2 — Reporting Summary [file 41467_2024_45254_MOESM2_ESM.pdf]

nature portfolio

Corresponding author(s): Xinxin Zhang and Yong Gan

Last updated by author(s): Jan 18, 2024

Reporting Summary

Nature Portfolio wishes to improve the reproducibility of the work that we publish. This form provides structure for consistency and transparency in reporting. For further information on Nature Portfolio policies, see our Editorial Policies and the Editorial Policy Checklist.

Statistics

For all statistical analyses, confirm that the following items are present in the figure legend, table legend, main text, or Methods section.

n/a	Confirmed	
☐	☒	The exact sample size (n) for each experimental group/condition, given as a discrete number and unit of measurement
☐	☒	A statement on whether measurements were taken from distinct samples or whether the same sample was measured repeatedly
☐	☒	The statistical test(s) used AND whether they are one- or two-sided<br>*Only common tests should be described solely by name; describe more complex techniques in the Methods section.*
☒	☐	A description of all covariates tested
☐	☒	A description of any assumptions or corrections, such as tests of normality and adjustment for multiple comparisons
☐	☒	A full description of the statistical parameters including central tendency (e.g. means) or other basic estimates (e.g. regression coefficient) AND variation (e.g. standard deviation) or associated estimates of uncertainty (e.g. confidence intervals)
☐	☒	For null hypothesis testing, the test statistic (e.g. F, t, r) with confidence intervals, effect sizes, degrees of freedom and P value noted<br>*Give P values as exact values whenever suitable.*
☒	☐	For Bayesian analysis, information on the choice of priors and Markov chain Monte Carlo settings
☒	☐	For hierarchical and complex designs, identification of the appropriate level for tests and full reporting of outcomes
☒	☐	Estimates of effect sizes (e.g. Cohen's d, Pearson's r), indicating how they were calculated

Our web collection on statistics for biologists contains articles on many of the points above.

Software and code

Policy information about availability of computer code

Data collection	Data were collected using the software described in each experiment.<br>The size and zeta potential data of nanovesicles were collected using Zetasizer Nano software (v 3.30). The fluorescence intensity and ultraviolet absorption were collected using BioTek Gen5 software (v 2.0). The confocal laser scanning microscopy images were captured using Olympus FV10-ASW Viewer software (v 4.2) and Leica Application Suite software (v 3.5). The data of flow cytometry was collected by CellQuest software. The biodistribution of nanovesicles in vivo and in major organs was imaged using IVIS spectrum system (Perkin Elmer, USA) with Living Image Software (v 4.0). ITC profiles were collected by ITC 200 software (v 1.26.1). The images of gels, dot blots and western blots were collected using a Bio-Rad ChemiDoc MP Imaging System, Tanon Camera Driver (v 3.3) and Tanon App for Biology (v 1.0).
Data analysis	Graphpad Prism (v 8.0) was used for statistical analysis; Image J (v 1.8.0) was used for image analysis; ChemBioDraw Ultra 12.0 was used for chemical formula; MestReNova (v 12.0.0) was used for proton nuclear magnetic resonance (1H-NMR) data analysis; OriginPro (v 9.1) was used to analyze ITC profiles. The data of flow cytometry was analyzed by FlowJo (v 7.6.1). 3dx Max 2014 (v 16.0) was used for the drawing of schematic diagrams.

For manuscripts utilizing custom algorithms or software that are central to the research but not yet described in published literature, software must be made available to editors and reviewers. We strongly encourage code deposition in a community repository (e.g. GitHub). See the Nature Portfolio guidelines for submitting code & software for further information.

Data

Policy information about availability of data

All manuscripts must include a data availability statement. This statement should provide the following information, where applicable:

- Accession codes, unique identifiers, or web links for publicly available datasets
- A description of any restrictions on data availability
- For clinical datasets or third party data, please ensure that the statement adheres to our policy

The authors declare that all data needed to support the finding of this study are presented in the Article, Supplementary information and Source data file. A reporting summary for this article is available as a Supplementary Information file. Source data are provided with this paper (figshare DOI: https://doi.org/10.6084/m9.figshare.24612237).

Research involving human participants, their data, or biological material

Policy information about studies with human participants or human data. See also policy information about sex, gender (identity/presentation), and sexual orientation and race, ethnicity and racism.

Reporting on sex and gender	Not available.
Reporting on race, ethnicity, or other socially relevant groupings	Not available.
Population characteristics	Not available.
Recruitment	Not available.
Ethics oversight	Not available.

Note that full information on the approval of the study protocol must also be provided in the manuscript.

Field-specific reporting

Please select the one below that is the best fit for your research. If you are not sure, read the appropriate sections before making your selection.

☒ Life sciences ☐ Behavioural & social sciences ☐ Ecological, evolutionary & environmental sciences

For a reference copy of the document with all sections, see nature.com/documents/nr-reporting-summary-flat.pdf

Life sciences study design

All studies must disclose on these points even when the disclosure is negative.

Sample size	Sample sizes were determined based on estimates from pilot experiments, as statistical methods were not used for sample size calculation. For in vitro experiments, at least triplicates were included to enable statistical calculations. For in vivo studies, a sample size of 3-6 animals per treatment group was deemed sufficient to reliably detect statistically significant differences.
Data exclusions	No data were excluded from the analyses.
Replication	All of the experimental findings could be reliably reproduced. All experiments were replicated multiple times with reproducible results as indicated in the figure legends. In animal experiments, at least six rats were included in each group.
Randomization	Cells or mice were randomly assigned to different groups before treatment.
Blinding	In the experiments of in vivo antitumor efficacy, different operators perform dosing and data collection. The investigators collecting and analyzing data were blinded to group allocation during data analysis. For other experiments, the investigators maintained awareness of the experimental conditions throughout the data acquisition and analysis process, and the investigators were unbiased in both outcome assessment and conclusion drawing.

Reporting for specific materials, systems and methods

We require information from authors about some types of materials, experimental systems and methods used in many studies. Here, indicate whether each material, system or method listed is relevant to your study. If you are not sure if a list item applies to your research, read the appropriate section before selecting a response.

Materials & experimental systems

n/a	Involved in the study
	☒ Antibodies
	☒ Eukaryotic cell lines
☒	Palaeontology and archaeology
	☒ Animals and other organisms
☒	Clinical data
☒	Dual use research of concern
☒	Plants

Methods

n/a	Involved in the study
☒	ChIP-seq
	☒ Flow cytometry
☒	MRI-based neuroimaging

Antibodies

Antibodies used	All the antibodies were diluted and used following the supplier's protocols.<br>Anti-IgG (Abcam, clone: RIGG-69, ab133470, 1:5000 dilution); anti-IgM (Abcam, clone: KT95, ab170492, 1:2000 dilution); HRP-conjugated anti-rabbit secondary antibody (Beyotime Biotechnology, A0208, 1:1000 dilution); anti-albumin (Abcam, clone: EPR20195, ab207327, 1:2000 dilution); Anti-F4/80 (Abcam, clone: EPR26545-166, ab300421, 1:100 dilution); anti-integrin beta 1 (Abcam, clone: EPR16896, ab179472, 1:1000 dilution); anti-OPN (Abcam, clone: RM1018, ab283656, 1:1000 dilution); anti-CD44 (Abcam, clone: EPR18668, ab189524, 1:1000 dilution); Alexa Fluor 647-labeled second antibody (Yeasen Biotechnology, 34213ES60, 1:200 dilution).
Validation	All antibodies used in this manuscript were commercially available. The validation and quality control are performed by the corresponding vendors, and available on the manufactures' website and datasheet.<br>Anti-IgG<br>https://www.abcam.cn/products/primary-antibodies/igg-antibody-rigg-69-ab133470.html<br>Anti-IgM<br>https://www.abcam.cn/products/primary-antibodies/hrp-igm-antibody-kt95-ab170492.html<br>Anti-albumin<br>https://www.abcam.cn/products/primary-antibodies/albumin-antibody-epr20195-ab207327.html<br>Anti-F4/80<br>https://www.abcam.cn/products/primary-antibodies/f480-antibody-epr26545-166-ab300421.html<br>Anti-OPN<br>https://www.abcam.cn/products/primary-antibodies/osteopontin-antibody-rm1018-ab283656.html<br>Anti-integrin beta 1<br>https://www.abcam.cn/products/primary-antibodies/integrin-beta-1-antibody-epr16896-ab179472.html<br>Anti-CD44<br>https://www.abcam.cn/products/primary-antibodies/cd44-antibody-epr18668-ab189524.html<br>HRP-conjugated anti-rabbit secondary antibody<br>https://www.beyotime.com/product/A0208.htm<br>Alexa Fluor 647-labeled second antibody<br>https://www.yeasen.com/products/detail/120

Eukaryotic cell lines

Policy information about cell lines and Sex and Gender in Research

Cell line source(s)	The human nonsmall cell lung cancer cell line A549, mouse mononuclear macrophage J774, human umbilical vein endothelial cells HUVEC and human cervical cancer cell HeLa were obtained from the Shanghai Institutes for Biological Sciences.
Authentication	Cell lines were authenticated by morphology, STR profiling, and PCR assays with species-specific primers.
Mycoplasma contamination	Cell lines were tested negative for mycoplasma contamination.
Commonly misidentified lines (See ICLAC register)	No commonly misidentified cell lines were used.

Animals and other research organisms

Policy information about studies involving animals; ARRIVE guidelines recommended for reporting animal research, and Sex and Gender in Research

Laboratory animals	6-8 weeks male Sprague-Dawley (SD) rats (200-200g), 4-6 weeks male and female BALB/c nude mice, and 4-6 weeks ICR mice were provided by the Animal Experiment Center of Shanghai Institute of Materia Medica (Shanghai, China), Chinese Academy of Sciences.
Wild animals	This study did not involve wild animals.
Reporting on sex	Male SD rats, and male BALB/c nude mice were used to investigate the pharmacokinetics and biodistribution of nanovesicles. Male

	ICR mice were used to analyze the level of anti-PEG. Male BALB/c nude mice were used to establish A549 tumor models for study of in vivo antitumor efficacy. Female BALB/c nude mice were used to establish HeLa tumor models for study of in vivo antitumor efficacy. We have provided the species, strain (including substrain), sex, number and age of animals in every experiment in the Methods. In Source data file, we have indicated the sex of animals for data collecting.
Field-collected samples	The study did not involve samples collected from the field.
Ethics oversight	All animal experiments were conducted following the relevant requirements of the Institutional Animal Care and Use Committee (IACUC) guidelines of the Shanghai Institute of Materia Medica, Chinese Academy of Sciences.

Note that full information on the approval of the study protocol must also be provided in the manuscript.

Plants

Seed stocks	Not available.
Novel plant genotypes	Not available.
Authentication	Not available.

Flow Cytometry

Plots

Confirm that:

- [x] The axis labels state the marker and fluorochrome used (e.g. CD4-FITC).
- [x] The axis scales are clearly visible. Include numbers along axes only for bottom left plot of group (a 'group' is an analysis of identical markers).
- [x] All plots are contour plots with outliers or pseudocolor plots.
- [x] A numerical value for number of cells or percentage (with statistics) is provided.

Methodology

Sample preparation	The sample preparation was provided under "Methods" section.<br>The DiO-labeled nanovesicles were incubated with cells, and collected for flow cytometry analysis to investigate the cellular uptake of nanovesicles in cells.
Instrument	The samples were detected using a BD Biosciences FACScalibur flow cytometer.
Software	The data was analyzed by Flowjo (v 7.6.1) software.
Cell population abundance	The cells used in the study were commercialized cells, with high abundance of cell population.
Gating strategy	Flow cytometry analysis in this study was used to investigate the cellular uptake of nanovesicles in cells, without necessary for gating by negative and positive samples.

- [x] Tick this box to confirm that a figure exemplifying the gating strategy is provided in the Supplementary Information.
